# Isolation, characterization and molecular cloning of Duplex-Specific Nuclease from the hepatopancreas of the Kamchatka crab

**DOI:** 10.1186/1471-2091-9-14

**Published:** 2008-05-21

**Authors:** Veronika E Anisimova, Denis V Rebrikov, Dmitry A Shagin, Valery B Kozhemyako, Natalia I Menzorova, Dmitry B Staroverov, Rustam Ziganshin, Laura L Vagner, Valery A Rasskazov, Sergey A Lukyanov, Alex S Shcheglov

**Affiliations:** 1 M.M. Shemiakin and Yu. A. Ovchinnikov Institute of Bioorganic Chemistry RAS, Miklukho-Maklaya 16/10, 117871 Moscow, Russia; 2 Evrogen JSC, Miklukho-Maklaya 16/10, 117871 Moscow, Russia; 3 Pacific Institute of Bioorganic Chemistry, RAS Far East Division, 690022 Vladivostok, Russia

## Abstract

**Background:**

Nucleases, which are key components of biologically diverse processes such as DNA replication, repair and recombination, antiviral defense, apoptosis and digestion, have revolutionized the field of molecular biology. Indeed many standard molecular strategies, including molecular cloning, studies of DNA-protein interactions, and analysis of nucleic acid structures, would be virtually impossible without these versatile enzymes. The discovery of nucleases with unique properties has often served as the basis for the development of modern molecular biology methods. Thus, the search for novel nucleases with potentially exploitable functions remains an important scientific undertaking.

**Results:**

Using degenerative primers and the rapid amplification of cDNA ends (RACE) procedure, we cloned the Duplex-Specific Nuclease (DSN) gene from the hepatopancreas of the Kamchatka crab and determined its full primary structure. We also developed an effective method for purifying functional DSN from the crab hepatopancreas. The isolated enzyme was highly thermostable, exhibited a broad pH optimum (5.5 – 7.5) and required divalent cations for activity, with manganese and cobalt being especially effective. The enzyme was highly specific, cleaving double-stranded DNA or DNA in DNA-RNA hybrids, but not single-stranded DNA or single- or double-stranded RNA. Moreover, only DNA duplexes containing at least 9 base pairs were effectively cleaved by DSN; shorter DNA duplexes were left intact.

**Conclusion:**

We describe a new DSN from Kamchatka crab hepatopancreas, determining its primary structure and developing a preparative method for its purification. We found that DSN had unique substrate specificity, cleaving only DNA duplexes longer than 8 base pairs, or DNA in DNA-RNA hybrids. Interestingly, the DSN primary structure is homologous to well-known Serratia-like non-specific nucleases structures, but the properties of DSN are distinct. The unique substrate specificity of DSN should prove valuable in certain molecular biology applications.

## Background

A large number of nucleases have been described in the literature. Among these are restriction endonucleases that target specific DNA sequences, as well as DNA-RNA non-specific endonucleases that hydrolyze nucleic acids and nucleoside monophosphates [1]. These enzymes are key components of many biologically important processes, including DNA replication, repair and recombination (including transposition), and antiviral defense, apoptosis and digestion. Because of their versatility and specificity, these enzymes have revolutionized molecular biology, making possible a host of modern techniques, including molecular cloning, studies of DNA-protein interaction and determination of nucleic acid structures. Because the discovery of nucleases with unique properties has often led to the development of new molecular biology methods, the search for novel nucleases remains an important scientific endeavor.

Here we present the results of our investigations on Duplex-Specific Nuclease (DSN) isolated from the hepatopancreas of the Kamchatka crab (*Paralithodes camtschaticus*). We cloned the gene encoding this enzyme, developed an enzyme purification method and determined some of the salient properties of the purified protein. DSN is thermostable, exhibiting a high optimal temperature, and specifically hydrolyzes double-stranded (ds) DNA or the DNA strand of DNA-RNA hybrids. These unique properties make this enzyme useful for a number of molecular biology applications, including full-length cDNA library normalization [2,3] or subtraction [4], genomic single-nucleotide polymorphism detection [5,6] and quantitative telomeric overhang determination [7]. The primary structure of DSN is homologous to that of Serratia-like non-specific DNA-RNA endonucleases, which hydrolyze both RNA and single-stranded (ss) and ds DNA. These results provide the first evidence that enzymes belonging to this family of non-specific DNA-RNA endonucleases may specifically target ds DNA-containing substrates.

## Results

### Cloning and expression of the DSN gene

**Figure 1:**
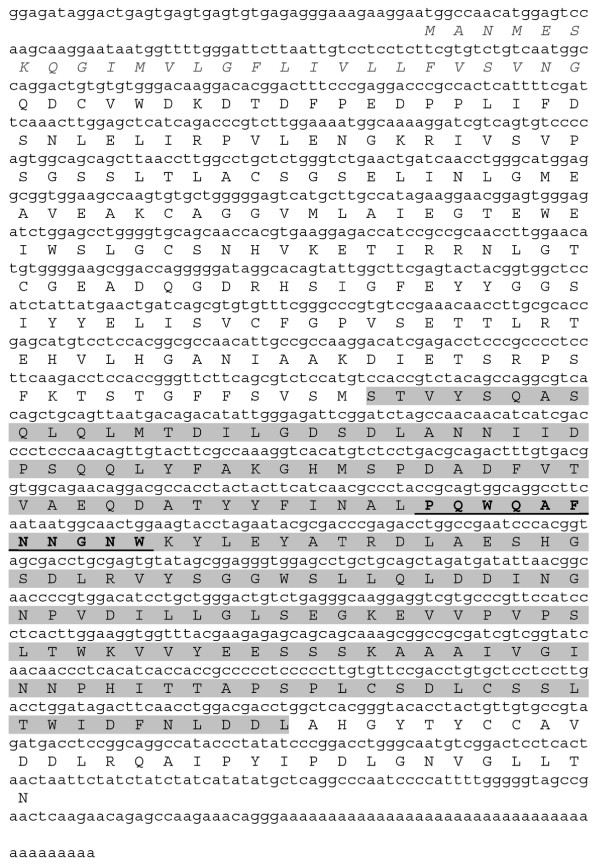
**Nucleotide and amino-acid sequences of DSN**. The NUC domain is colored gray. The sequence that was utilized to synthesize degenerated oligonucleotides is displayed as bold and underlined. The predicted signal peptide is italicized.

To clone the DSN gene, we used degenerate oligonucleotides corresponding to the most conserved region of the kuruma shrimp (*Marspenaus japonicus*) nuclease amino acid sequence [CAB55635]. Applying the rapid amplification of cDNA ends (RACE) procedure, we amplified the 3'-terminus of DSN cDNA to obtain a 600-base-pairs (bp) PCR product. This cDNA fragment was cloned and sequenced. Using the resulting nucleotide sequence, we synthesized several specific oligonucleotides for rapid amplification of DSN cDNA 5' ends. Amplification proceeded in three stages, leading to the isolation of a full-length DSN cDNA comprising 1348 nucleotides, excluding the poly(A) tail (Figure 1). Analysis of this sequence revealed a 1221-nucleotide open reading frame (starting with the first initiator codon) that encoded a 407-amino acid protein (Figure 1). The Kamchatka crab DSN was very similar to the kuruma shrimp nuclease, exhibiting 67% amino-acid identity and 82% overall homology, taking conservative substitutions into account.

According to the SMART program [8], residues 1–26 of DSN form a signal sequence that allows for secretion, whereas residues 195–376 form a NUC domain characteristic of Serratia DNA-RNA non-specific endonucleases (Figure 1). The predicted mature protein had a molecular mass of 41.5 kDa and an isoelectric point of 4.2.

We cloned DSN into the pET22b(+) expression vector, which is typically used to express secretory or toxic proteins. This vector promotes expression of recombinant proteins with an *E. coli*-specific N-terminal signal sequence that initiates protein secretion into the periplasmic space, and contains a C-terminal hexa-histidine sequence that allows purification of the enzyme using metal-affinity chromatography. The cloned DSN cDNA lacked non-translated regions and was also likely missing the region that encoded the signal peptide. Based on SDS-polyacrylamide gel electrophoresis (SDS-PAGE) the recombinant protein was present only in inclusion bodies of *E. coli*, and as isolated, was inactive (see Additional file 1). We subsequently dissolved inclusion bodies in 8 M urea and isolated recombinant DSN using metal-affinity chromatography under denaturing conditions (see Additional file 1).

### Isolation of DSN from the Kamchatka crab hepatopancreas

Before developing a procedure for purifying DSN from the crab hepatopancreas, we first developed anti-DSN antibodies to allow us to follow DSN through the different stages of the purification process. For this purpose, we purified recombinant DSN from *E. coli *using denaturing metal-affinity chromatography (see Methods) and used it to immunize rabbits. After a series of four injections, we collected blood and analyzed the resulting sera. Using Western blot hybridization, we titrated the antibody and found that at a dilution of 1/5,000 the anti-serum was highly specific for both natural and recombinant DSN.

**Figure 2:**
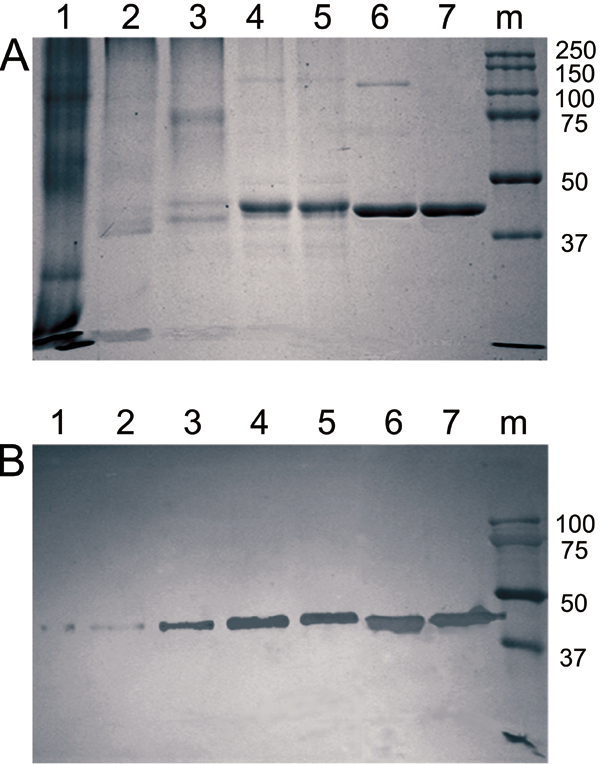
**Purification of DSN from the crab hepatopancreas**. **A: **Composition of protein samples obtained at different stages of DSN purification, determined by SDS-PAGE and Coomassie R-250 staining. **B: **Composition of protein samples obtained at different stages of DSN purification, determined by SDS-PAGE and immunoblotting.**Lanes: **1, crude extract; 2, fractions obtained after DEAE MacroPrep chromatography; 3 and 4, combined fractions after first and second Phenyl Sepharose chromatography steps; 5, fractions obtained after hydroxyapatite chromatography; 6, fractions obtained after Heparin Sepharose chromatography; 7, final preparation obtained after Sephacryl S200 chromatography. M, molecular mass marker with molecular mass (in kDa) indicated at right.

A combination of different chromatographic methods was used to isolate DSN from the crab hepatopancreas. All stages of purification were followed by SDS-PAGE with Coommasie staining (Figure 2A) and immunoblotting (Figure 2B), and the specific enzymatic activity of fractions obtained was determined by a modified Kunitz method. Results from a typical DSN purification procedure are presented in Table 1. Anion-exchange chromatography on DEAE MacroPrep columns resulted in approximately a 5-fold increase in specific activity. The protein obtained at this stage was subjected to two consecutive rounds of chromatography on Phenyl Sepharose, resulting in approximately a 100-fold purification. At this point in the purification process, most of the pigment present in the original sample had been removed. Hydroxyapatite chromatography was used to remove excess NaCl present in the protein sample after hydrophobic chromatography. Although this step had little effect on protein purity, it was useful for subsequent the Heparin Sepharose chromatography step (see Additional file 2), which was very effective, increasing DSN purification by ~5-fold (Table 1). Gel-filtration performed as the last stage of purification resulted in a single symmetric peak possessing high specific nuclease activity (see Additional file 3). SDS-PAGE of the protein eluting in this peak yielded a single band that reacted with the polyclonal antibody raised against recombinant DSN (Figure 2). The apparent molecular mass determined by size-exclusion chromatography (~40 kDa) was in good agreement with the apparent molecular mass determined by SDS-PAGE (~44 kDa), indicating that DSN was present in the form of a monomer.

**Table 1:** Typical results of DSN purification from crab hepatopancreas.

Stage of purification	Total protein, mg	Specific activity, Kunitz/mg	Activity yield, %	Fold purification
Kamchatka crab hepatopancreas extract	1948.0	51	100	1.0
Chromatography on DEAE MacroPrep	275.2	265	73	5.2
Chromatography on Phenyl Sepharose	70.0	801	56	15.7
Re-chromatography on Phenyl Sepharose	10.0	4900	49	96.1
Chromatography on Hydroxyapatite	8.1	5200	43	102.0
Chromatography on Heparin Sepharose	1.4	29000	40	568.6
Gel filtration on Sephacryl S-200	0.95	31500	30	617.7

### Mass-spectrometric analysis of native DSN

Digestion of DSN with trypsin generated set of peptides. Four of them were identified by parallel mass-spectrometry (MS) as DIETSRPSFK (score 54), YLEYATR (score 31), DLAESHGSDLR (score 73), and EVVPVPSLTWK (score 58). Individual ion scores > 51 indicate identity or extensive homology (p < 0.05). Each of these sequences was present in DSN, and no other proteins containing more than one of those peptides were identified in non-redundant protein databases.

### Some properties of Kamchatka crab hepatopancreatic DSN

#### Stability and physico-chemical properties

DSN is a highly stable enzyme, losing only part of its activity after heating at 70°C for 30 min. Even DSN incubated for 30 min at 100°C retained 7% of its activity. DSN was resistant to proteinase K (at 37°C) and remained active over a very wide range of pH values (4–12). Incubating for 1 hr at 37°C in the presence of 1% SDS, 10 mM mercaptoethanol or 0.3% hydrogen peroxide resulted in only a 10% decrease in specific activity. However, a 1-hr incubation at 60°C in the presence of 1% SDS, 10 mM mercaptoethanol or 0.3% hydrogen peroxide decreased specific activity by 80 to 90%.

#### Effect of different reaction conditions on DSN activity

**Figure 3:**
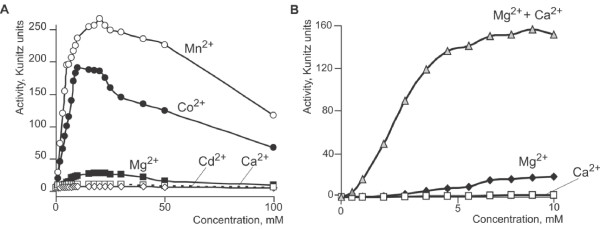
**A: **Relationship between divalent cation concentration and DSN activity. The incubation mixtures (50 mM Tris-HCl, pH 7.15) contained calf thymus DNA (50 μg/ml), 8 units/ml DSN and different concentrations of MnCl_2_, CoCl_2_, MgCl_2_, CdCl_2_, CaCl_2_. **B: **Relationship between DSN activity and concentration of magnesium chloride, calcium chloride and their mixture. The incubation mixtures (50 mM Tris-HCl, pH 7.15) contained calf thymus DNA (50 μg/ml), 8 units/ml DSN and different concentrations of MgCl_2_, CaCl_2_, and mixtures of MgCl_2 _and CaCl_2_.

DSN requires the presence of divalent cations in the reaction mixture for activity and was completely inhibited by EDTA (data not shown). Manganese chloride (MnCl_2_) and cobalt chloride (CoCl_2_) activated DSN very effectively, whereas magnesium chloride (MgCl_2_), calcium chloride (CaCl_2_) or cadmium chloride (CdCl_2_) were significantly less effective (Figure 3A). The optimal ion concentration was ~20 mM; higher concentrations of divalent cations inhibited DSN activity.

We found that calcium chloride exerted a synergistic effect when added to reaction mixtures containing other cations. For example, simultaneous addition of 10 mM calcium chloride and 10 mM MgCl_2 _produced an increase in activity that was significantly higher than the total activity detected in the presence of any single cation (Figure 3B). No other cations possessed such synergistic activity.

**Figure 4:**
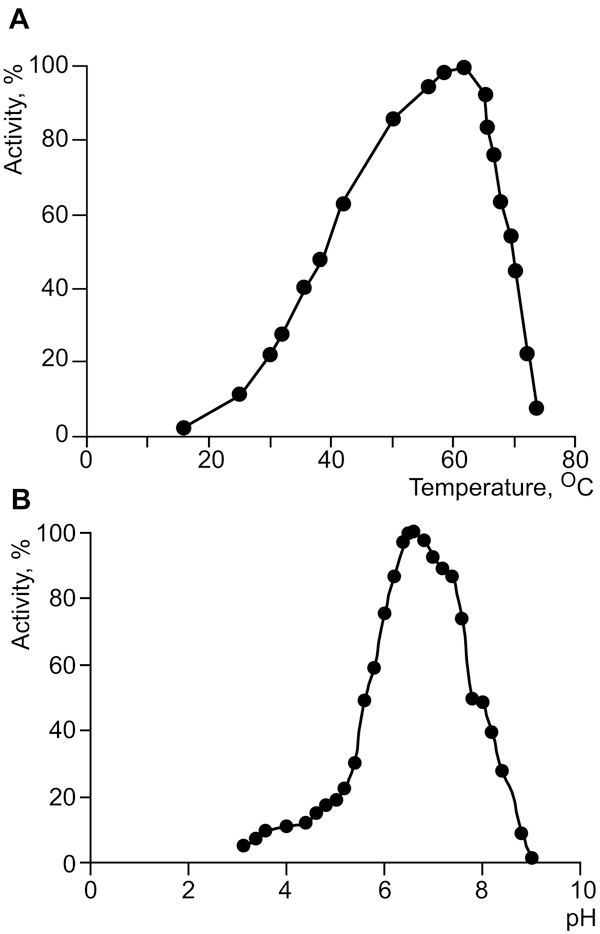
**A: **Effect of temperature on DSN activity. Calf thymus high-molecular-weight DNA (50 μg/ml) was dissolved in 50 mM Tris-HCl, pH 7.15 containing 5 mM MgCl_2 _and incubated with DSN (1 units/ml) for 2 min at different temperatures. **B: **Dependence of DSN activity on pH. Calf thymus high-molecular-weight DNA (50 μg/ml) was dissolved in 50 mM buffer containing 5 mM MgCl_2_. The pH was adjusted to the desired pH using glycine-HCl, sodium acetate, MES-NaOH, Tris-HCl and sodium borate buffers. The sample was equilibrated at 25°C and activity was measured using the Kunitz method.

The temperature optimum of DSN was determined using calf thymus DNA as a substrate. Under the conditions used, the highest activity was observed at ~60°C. Further increases in temperature were accompanied by a sharp decrease in enzymatic activity (Figure 4A). This might reflect enzyme denaturation, but was more likely due to melting of the double-stranded DNA substrate.

DSN has a very broad pH optimum (Figure 4B) with maximum located at ~pH 6.6. At pH values less than 3.0 or higher than 9.0, DSN was completely inactive. Increases in ionic strength were accompanied by decreases in DSN enzymatic activity. For example, addition of 0.2 M NaCl decreased enzymatic activity 10-fold. It is worth noting that urea was less effective in inhibiting enzymatic activity than was NaCl, requiring a concentration of 3 M to inhibit DSN activity by 10-fold.

As is the case with many other nucleases, DSN is sensitive to polyamines. For instance, spermidine at low concentrations (2–4 mM) slightly activated DSN, whereas at higher concentration (> 8 mM) significantly inhibited DSN DNAse activity (data not shown).

### Substrate specificity of DSN

**Figure 5:**
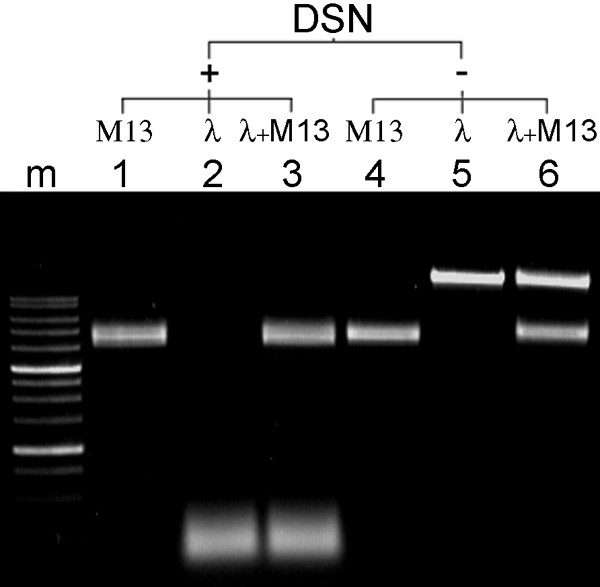
**DSN activity against high molecular weigh ss and ds DNA**. Agarose gel electrophoresis of M13 and lambda phage DNA before and after treatment for 10 min with 2 units of DSN at 70°C. **Lanes: **1, M13 phage DNA treated with DSN; 2, lambda phage DNA treated with DSN; 3, mixture of M13 phage and lambda phage DNA treated with DSN; 4, untreated M13 phage DNA; 5, untreated lambda phage DNA; 6, untreated mixture of M13 phage and lambda phage DNA; m, 1-kb ladder (SibEnzyme).

We investigated the effects of DSN on ss and ds DNA using M13 phage DNA and the lambda phage genome as ss and ds DNA substrates, respectively. We found that DSN hydrolyzed only ds DNA; ss DNA was left uncleaved (Figure 5). Notably, all these experiments were performed at high temperature (60–70°C). Taken together with the unusually high specificity of DSN and its thermostability we would expect that this enzyme could be used successfully for removing ds DNA from ss and ds DNA mixtures, for example, following hybridization.

**Figure 6:**
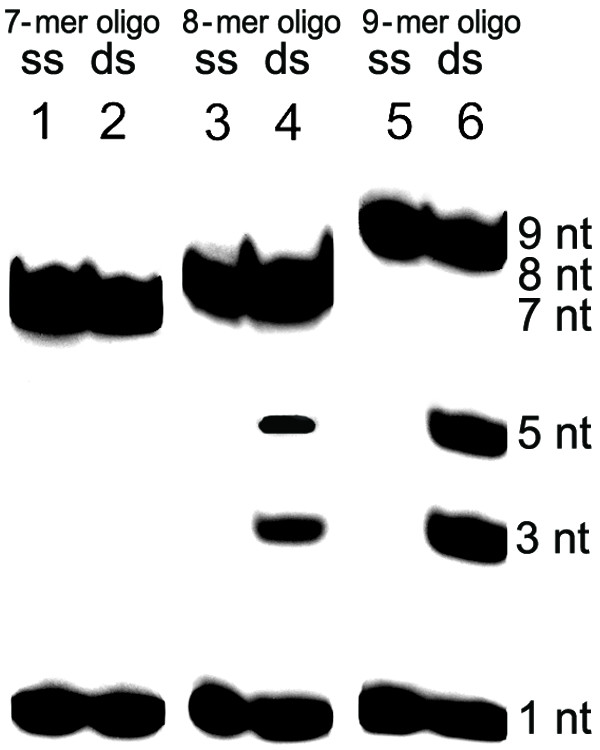
**DSN activity against synthetic DNA duplexes**. Each oligonucleotide (10 pM) was dissolved in 20 μl 25 mM Tris-HCl, pH 7.5 containing 5 mM MgCl_2 _and incubated with 1 Kunitz unit of DSN for 1 hour at 30°C. Reactions were stopped by adding EDTA to a final concentration of 5 mM. Products were separated by urea-polyacrylamide gel electrophoresis and analyzed autoradiographically by exposing to BioMax film (Kodak).**Lanes: **1, radiolabeled p7 (7-mer); 2, Na21T7 (43-mer) + p7; 3, radiolabeled p8 (8-mer); 4, Na21T7 + p8; 5, radiolabeled p9 (9-mer); 6, Na21T7 + p9.

We also evaluated DSN for DNA duplex length requirements. Radiolabeled oligonucleotides containing 7, 8 or 9 bases were hybridized with non-labeled 43-base oligonucleotide and incubated with DSN. We found that 7-bp duplexes were not cut, 8-bp duplexes were only slightly degraded and 9-bp duplexes were cleaved normally (Figure 6).

**Figure 7:**
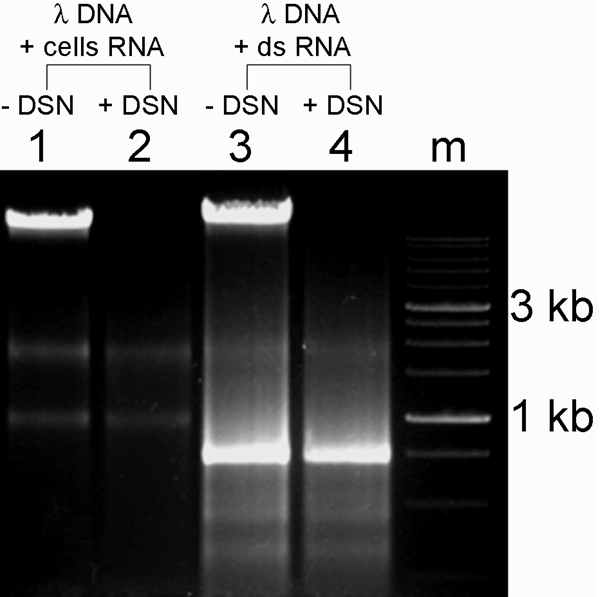
**DSN activity against ds and ss RNA**. Agarose gel electrophoresis of ds or ss RNA and ds DNA before and after treatment with DSN. **Lanes: **1, untreated lambda phage DNA (500 ng) and total RNA (50 ng) from cultured cells; 2, lambda phage DNA and total RNA after incubation with 2 units of DSN for 30 min at 37°C; 3, untreated lambda phage DNA and synthetic ds RNA (2 μg); 4, lambda phage DNA and synthetic ds RNA after incubation with 2 units of DSN for 30 min at 37°C; m, 1-kb ladder (SibEnzyme).

To analyze possible interactions of DSN with ss or ds RNA, we used total RNA isolated from cells as ss RNA and synthesized ds RNA *in vitro*. We found that DSN was unable to cleave either ss or ds RNA (Figure 7), suggesting that DSN would be very useful for removing DNA impurities during RNA isolation procedures.

**Figure 8:**
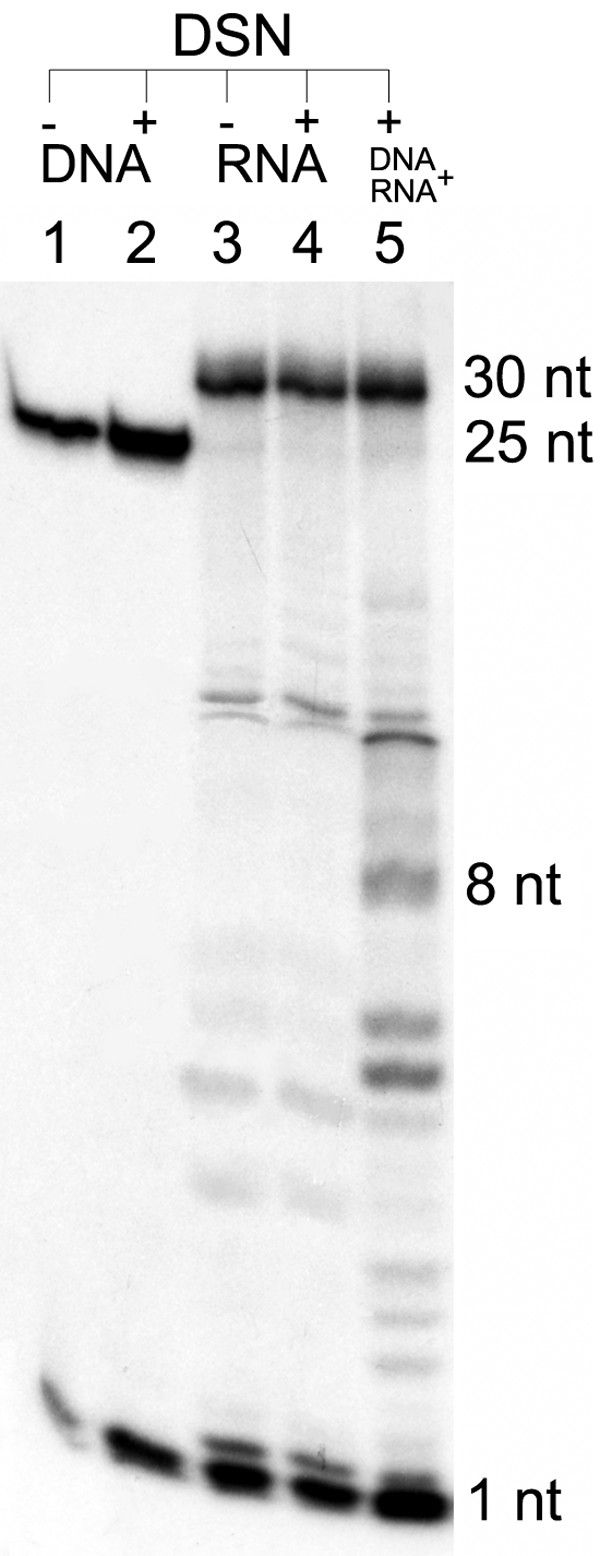
**DSN activity against synthetic DNA-RNA hybrids**. Ten picomoles of p25 DNA (25-mer) and 10 pM of ribo30 RNA (30-mer) were dissolved in 20 μl 25 mM Tris-HCl, pH 7.5 containing 5 mM MgCl_2 _and incubated with 1 Kunitz unit of DSN for 1 hr at 45°C. Reactions were stopped by adding EDTA to a final concentration of 5 mM. Products were separated by urea-polyacrylamide gel electrophoresis and analyzed autoradiographically by exposure to BioMax film (Kodak). **Lanes: **1, p25, untreated; 2, p25, treated with DSN: 3, ribo30, untreated; 4, ribo30, treated with DSN; 5, ribo30 + p25, treated with DSN.

Finally, the activity of DSN toward DNA-RNA hybrids was studied by hybridizing complementary deoxyribo- and ribo-oligonucleotides and digesting with DSN. Interestingly, the deoxyribo-oligonucleotide was completely degraded, but the ribo-oligonucleotide remained intact (Figure 8).

## Discussion

Our investigation into the structure and properties of the Kamchatka crab hepatopancreas DSN was prompted by the discovery of a novel nuclease from the kuruma shrimp hepatopancreas [9,10]. Initially described as a DNase [9], this shrimp nuclease has since been found to possess properties, including weak RNase activity [10], that are very different from those of bovine DNase I. A primary structure analysis showed that the kuruma shrimp nuclease was homologous to Serratia-like DNA-RNA non-specific endonucleases, which hydrolyze RNA as well as ss and ds DNA [1].

Investigations of invertebrate nucleases led to identification of a thermostable DNase in the hepatopancreas of the Kamchatka crab. This enzyme (or enzymes) was shown to predominantly hydrolyze ds DNA, but the structure and detailed properties of this enzyme remained uncharacterized [11]. To clone this enzyme, identified subsequently as a duplex-specific nuclease (DSN), we applied the RACE procedure using degenerate oligonucleotides homologous to the most conserved region of the shrimp nuclease. The sequence of cloned DSN was very similar to that of the shrimp endonuclease and contained an NUC-domain, characteristic of Serratia RNA/DNA non-specific endonucleases. Unfortunately, DSN expressed in *E. coli *was localized to inclusion bodies and could not be isolated in an active form.

Although our attempts to express functional recombinant protein were unsuccessful, we were able to use purified, denatured DSN to generate a highly specific antibody to aid in the development of a successful strategy for purifying DSN from the Kamchatka crab hepatopancreas. The method developed consisted of a series of chromatographic steps and resulted in a > 600-fold purification of the enzyme. After the final step, DSN had been purified to homogeneity and was found to possess a specific activity of 31,000 Kunitz per mg protein, and activity comparable to that of the corresponding shrimp nuclease. The identity of both the denatured recombinant protein (encoded by cloned DSN gene) and active DSN purified from the crab hepatopancreas was confirmed by mass-spectrometric peptide analysis and immunoblotting.

The activity of purified DSN depended on the presence of divalent cations, primarily manganese, cobalt and magnesium. Calcium did not activate DSN *per se *but significantly enhanced the activating effects of manganese, cobalt and magnesium ions. DSN is very stable; for example, incubation in the presence of β-mercaptoethanol, SDS and hydrogen peroxide at 37°C did not significantly decrease its activity. DSN was inactivated only at 70°C or higher, although even after boiling, DSN retained partial activity. The optimal temperature for DSN activity was approximately 60°C.

The substrate specificity of DSN appears to be unique among members of its family. DSN hydrolyzed only ds DNA or DNA in DNA/RNA hybrids regardless of nucleotide sequence and did not cleave ss DNA or any RNA. Moreover, DSN cleaved DNA duplexes containing at least nine base-pairs and left shorter DNA duplexes intact. To our knowledge, no enzyme with these particular specificities has been previously described in the literature.

The fact that DSN belongs to the Serratia-like family is of special interest. Although these non-specific DNA-RNA endonucleases share characteristic amino acids in their active center, some members of this group, notably DSN, possess unusually high specificity. The mechanism that underlies this fascinating duality warrants further investigation.

It is important to note that DSN combines high specificity and high stability. This property makes DSN an attractive tool for molecular biology applications. Prior to the discovery of DSN, there were no effective and simple methods for separating ss and ds DNA, which is a central issue in the normalization of cDNA libraries [12]. A number of important molecular methodologies have relied on the unique properties of DSN, including the original method for detecting point-nucleotide replacement mutations in a genome [5,6], the method for full-length cDNA library normalization [2,3] and subtraction [4], and quantitative telomeric overhang determination [7]. The application of DSN in the full-length cDNA libraries normalization procedure, in particular, has no competitive alternative.

## Conclusion

We have described a new duplex-specific nuclease from the Kamchatka crab hepatopancreas, determined its primary structure and developed a preparative method for its purification. We found that DSN had unique substrate specificities, cleaving only DNA duplexes longer than eight base-pairs, or DNA in DNA-RNA hybrids. Interestingly, the DSN primary structure is homologous to well-known non-specific nucleases structures, but its properties are quite different. The unique DSN substrate specificity makes it useful for a number of molecular biology applications.

## Methods

### cDNA first strand synthesis

RNA was isolated using TRIzol reagent (Invitrogen) according to manufacturer's instructions. cDNA first strand synthesis and cDNA library amplification were performed using the Super SMART PCR cDNA Synthesis Kit (Clontech) standard protocol with 1 μg total RNA and 20 pM poly dT primer [13]. Reverse transcriptase was inactivated after the reaction by heating at 95°C for 5 min.

Rapid amplification of DSN cDNA ends was performed using the SMART RACE cDNA Amplification Kit (Clontech) as described by the manufacturer [14,15]. The 3'-terminus was amplified using the degenerate oligonucleotides 5-CCTCAGTGGCA(G/A)GCTTT(C/T)AAT-3 and 5-CAGGCCTTTAATAATGG(T/A/G/C)AA(T/C)TGG-3 for the first and second PCR, respectively. Amplification was performed using the following cycling conditions: 94°C/20 sec., 64°C/30 sec., 72°C/60 sec. The 5'-terminal amplification step employed the same cycling conditions using the following specific oligonucleotides: 5'-GGCCAGGTCTCGGGTCGC-3', 5'-GGGTCGCGTATTCTAGGTA-3' and 5'-CCATTATTGAAGGCCTGCCA-3'. The resulting cDNA fragments were cloned into the pGEM-T vector (Promega) and sequenced.

### Amplification and cloning of DSN for expression

The DSN coding sequence was amplified using Kamchatka crab first strand cDNA as a template and the following oligonucleotides: 5'-TTG GAT CCT GTC AAT GGC CAG GAC TGT GTG TGG-3' and 5'-AA AAG CTT AGT GAG GAG TCC GAC ATT GCC CAG-3'. Amplification was performed in two stages: (1) 94°C/20 sec., 58°C/30 sec., 72°C/90 sec. (five cycles) and (2) 94°C/20 sec., 72°C/2 min. (25 cycles). The PCR product was purified using a Qia quick PCR purification kit (Qiagen) according to the manufacturer's instruction. The pET22b(+) vector and purified PCR products were then digested with the restriction enzymes, BamHI and EcoRI, alcohol precipitated and ligated. The *E. coli *NM522 strain (ampicillin-selected) was transformed with the resulting mixture. Clones were screened by PCR, and then plasmid DNA was isolated from positive clones and verified by sequencing. Recombinant enzyme was expressed in *E. coli *strain BL 21 (DE3) (ampicillin-selected).

### Cultivation of recombinant DSN-expressing E. coli

An overnight culture of producer strain cells cultivated in LB broth with ampicillin (200 mg/L) at 30°C was re-seeded at a 100-fold dilution into fresh LB medium with ampicillin and cultivated at 37°C for 2 hrs. IPTG was added to a final concentration of 1.0 mM and culturing was continued for 5 hrs at 30°C. The cells were collected by centrifugation and used for protein isolation.

### Isolation of recombinant DSN using cobalt-based sorbent

Cells collected from 50 ml cultures were resuspended in 0.05 M Tris-HCl, pH 8.0 and sonicated three times (1 min, 25 watt). After centrifugation at 10,000 × g for 15 min, the pellet was resuspended in 10 ml 0.05 M Tris-HCl buffer, pH 8.0 and sonicated. These procedures were repeated three times and the pellet thus obtained was solubilized by addition of 8 M urea in 50 mM Tris-HCl, pH 8.0 and clarified by centrifugation. The resulting solution was mixed with 1 ml 50% TALON suspension (Clontech) that had been previously washed with 0.05 M Tris-HCl buffer, pH 8.0 containing 8 M urea (Buffer A). The resulting suspension was incubated for 30 min at room temperature (or 4°C) with rotation and centrifuged at 1,000 × g for 2 min. The resulting pellet was washed five times with Buffer A and the proteins were eluted with Buffer A containing 100 mM EDTA.

### Determination of DNase activity

Two different methods were used to determine DNase activity. The first was a modified Kunitz method, which is based on the hyperchromic effect [16,17]. Briefly, calf thymus high-molecular-weight DNA (50 μg/ml) was dissolved in 50 mM Tris-HCl, pH 7.15 containing 5 mM MgCl_2_. The solution (1 ml) was equilibrated at 25°C and, after addition of sample to be analyzed, the optical density at 260 nm was measured every 5–10 sec. for 5 min. One unit of activity was taken as the quantity of enzyme that induced an increase of 0.001 A_260 _optical units/min. The second method was based on the accumulation of soluble DNA hydrolysis products. In this approach, 80 μl of an aqueous solution containing 40 μg calf thymus DNA was mixed with 10 μl reaction buffer (500 mM Tris-HCl, pH 8.0, and 100 mM MgCl_2_) and 10 μl of the solution under study. After incubating for 1 hr at 60°C, 10 μl 3 M potassium acetate buffer, pH 4.8 and 300 μl ethanol were added to the incubation mixture. After centrifugation, the supernatant was collected and brought to 2 ml with water. The optical density at 260 nm was measured against a control sample without enzyme.

### Polyclonal antibodies against recombinant DSN

DSN isolated under denaturating conditions was precipitated by trichloroacetic acid and the pellet was washed three times with acetone. The DSN sample thus obtained was resuspended in complete Freund's adjuvant and injected into yearling rabbit four times within three months (1 mg/injection). Serum was collected and used as the source of polyclonal antibodies.

### Purification of DSN from the Kamchatka crab hepatopancreas

All procedures were performed at 4°C. Hepatopancreas (100 g) collected from freshly captured king crabs was suspended in 200 ml of 50 mM Tris-HCl, pH 7.15 buffer and homogenized (Polytron). After centrifugation at 12,000 × g for 30 min, the supernatant was applied to a DEAE MacroPrep column (2.5 cm × 20 cm) equilibrated with 50 mM Tris-HCl, pH 7.15. The column was washed with four column-volumes of the same buffer and the proteins were eluted using stepwise increases in NaCl concentration (0.4, 0.5 and 1 M). All operations were performed at a flow-rate of 3 ml/min. The fractions were analyzed by SDS-PAGE and immunoblotting with anti-DSN antibody, and by determining the specific DNAse activity of fractions, as described above. DSN-enriched fractions with high specific activity were collected and brought to 5 M NaCl by adding solid NaCl.

The sample was loaded onto a Phenyl-Sepharose (Sigma) column (2.5 cm × 30 cm) equilibrated with 50 mM Tris-HCl, pH 7.15 containing 5 M NaCl. The column was washed with four column-volumes of the same buffer, and the proteins were eluted by stepwise decreases in NaCl concentration (4.5, 4.0 and 3.5 M). DSN-enriched fractions with high specific activity were collected, brought to 5 M with solid NaCl and re-chromatographed on Phenyl-Sepharose under the same conditions.

DSN-enriched fractions with high specific activity were collected and loaded onto a hydroxyapatite (Bio-Rad) column (2.5 cm × 20 cm) equilibrated with 50 mM Tris-HCl, pH 7.15. The proteins were eluted by stepwise increases (10, 25 and 250 mM) of sodium phosphate buffer, pH 7.5.

DSN-enriched fractions with high specific activity were collected and applied to a Heparin-Sepharose (Sigma) column (1.5 cm × 20 cm) equilibrated with 50 mM Tris-HCl, pH 7.15. The proteins were eluted by stepwise increases (0.05, 0.1 and 1.0 M) in NaCl concentration. A flow rate of 1.5 ml/min was used throughout.

DSN-enriched fractions with high specific activity were collected and concentrated to 0.1 ml on UltraFree 15 and UltraFree 0.5 Biomax 5K (Millipore) centrifugal columns and applied to a Sephacryl S-200 (Amersham-Pharmacia) column (1 cm × 70 cm) equilibrated with 50 mM Tris-HCl, pH 7.5, containing 150 mM NaCl. All operations were performed at a flow rate of 0.3 ml/min. The fractions containing DSN were combined, desalted and concentrated on UltraFree 15 and UltraFree 0.5 Biomax 5K (Millipore) centrifugal columns. Glycerol was added to 50% and the samples were stored at -20°C.

Protein concentration was determined using the Bradford method [18].

### Mass-spectrometric analysis of native DSN

Isolated DSN was digested in-gel after SDS-PAGE using trypsin, as described previously [19]. Mass-spectrometric analysis was performed using a MALDI-TOF/TOF with LIFT capability (Ultraflex TOF/TOF, Bruker Daltonik GmbH, Bremen, Germany). Ionization was performed with a 337-nm pulsed nitrogen laser. MS/MS experiments on the four most intensive peptide peaks of the digested protein were performed by means of laser-induced dissociation (LID). All data were processed using the FlexAnalysis version 2.4 and BioTools version 3.0 Software packages (Bruker Daltonics GmbH). Fragmentation information from the multiple LID spectra combined with TOF spectra was submitted to the sequence query program, Mascot [20], for possible identities, using a precision tolerance of 0.1 Da for the parent peptide and 0.5 Da for the MS/MS fragments, and the following search parameters: genome database – NCBI; taxonomy – All; enzyme – trypsin.

### Determination of optimal conditions for DSN enzymatic activity

DSN activity was determined in incubation mixtures containing different concentrations (0–100 mM) of individual divalent cations (Mg^+2^, Ca^+2^, Mn^+2^, Co^+2^, Cd^+2^) or cation mixtures as indicated in figure legends. Incubations were performed at temperatures between 25 and 85°C in sealed tubes to prevent evaporation. Activity was measured at pH values ranging from 3 to 11, adjusted using 50 mM glycine-HCl, sodium acetate, MES-NaOH, Tris-HCl and sodium borate buffers. Optimal conditions were determined using the Kunitz method. Potential inhibitory or stimulatory effects of factors added to incubation mixtures were similarly tested.

### Investigation of DSN stability

DSN was incubated from 25 to 100°C (pH 7.5), at pH values ranging from 0 to 14 (25°C), or in the presence of dithiothreitol, mercaptoethanol, SDS or hydrogen peroxide (pH 7.5, 37 or 60°C). DSN activity under each condition was compared with that obtained under optimal conditions using the Kunitz method.

### Investigation of DSN substrate specificity

DSN substrate specificity was investigated by analyzing the degradation of high-molecular-weight nucleic acids. Reactions with M13 phage DNA (100 ng) and lambda phage DNA (500 ng) were performed at 70°C; reactions with RNA were performed at 37°C. DSN was incubated with nucleic acids, dissolved in 50 mM Tris-HCl, pH 7.5 containing 5 mM MgCl_2 _in the total volume of 30 μl. Reactions were stopped by adding EDTA to a final concentration of 5 mM. Nucleic acid fragmentation was analyzed by agarose gel electrophoresis with ethidium bromide staining.

The following synthetic oligonucleotides were also used as substrates: Na21T7 (5'-TGTAGCGTGAAGACGACAGAAGTAATACGACTCACTATAGGGC-3', 43-mer), p7 (5'-GCCCTAT-3', 7-mer), p8 (5'-GCCCTATA-3', 8-mer), p9 (5'-GCCCTATAG-3', 9-mer), p25 (5'-CCCGCCCATCCGTGAGTCGTATTAG-3', 25-mer), and ribo30 (ribo-oligonucleotide, 5'-CTAATACGACTCACGGATGGGCGGGAATAA-3', 30-mer). All oligonucleotides, with the exception of Na21T7, were radiolabeled with γ-^32^P rATP using polynucleotide kinase (SibEnzyme).

Reactions with DNA duplexes were performed at 30°C; reactions with DNA-RNA hybrids were performed at 45°C. DSN (1 Kunitz unit) was incubated for 1 hr with oligonucleotides (10 pM each) dissolved in 20 μl 25 mM Tris-HCl, pH 7.5 containing 5 mM MgCl_2 _in the total volume of 30 μl. Reactions were stopped by adding EDTA to a final concentration of 5 mM. DSN-mediated digestion of oligonucleotides was analyzed by urea-polyacrylamide gel electrophoresis followed by autoradiography (BioMax film, Kodak).

## Abbreviations

DSN: duplex-specific nuclease; ds: double stranded; ss: single stranded; nt: nucleotides; NUC domain: DNA-RNA non-specific endonuclease domain; RACE: rapid amplification of cDNA ends; bp: base pairs; SDS-PAGE: SDS-polyacrylamide gel electrophoresis; EDTA: ethylenediaminetetraacetate; MS: mass-spectra.

## Authors' contributions

VEA carried out DSN expression and characterization; DVR carried out cloning procedures; DAS carried out DSN substrate-specificity experiments with oligonucleotides; DBS prepared antibodies against DSN; VBK and NIM carried out crab hepatopancreas preparation and preliminary DSN purification experiments; RZ carried out DSN mass-spectrometric analyses; LLV carried out RNA isolation and cDNA synthesis, VAR and SAL conceived of the study, participated in its design and coordination, ASS carried out DSN purification and prepared manuscript. All authors read and approved the final manuscript.

## Supplementary Material

Additional file 1 Additional figure 1. DSN expression in E. coli. SDS-PAGE of protein fractions before and after purification by metal-affinity chromatography. Cont lanes: protein samples obtained from control E. coli strains containing pET; Exp lanes: proteins obtained from E. coli strains expressing pET-DSN. Lane 1 and 2, total protein; lane 3 and 4, protein compositions of inclusion bodies; lane 5 and 6, proteins purified from inclusion bodies by metal-affinity chromatography; lanes 7 and 8, proteins purified by metal-affinity chromatography of soluble fractions. M denotes the markers. Molecular masses of standards are indicated at left. Click here for file

Additional file 2 Additional figure 2. Elution profile of DSN on Heparin Sepharose. The heparin Sepharose column (1.5 × 20 cm) was equilibrated with 50 mM Tris-HCl, pH 7.15. Proteins were eluted using stepwise increases in NaCl concentration (50 mM, 100 mM and 1 M) at a flow rate of 1.5 ml/min. Fractions with DNAse activity are indicated in gray. Click here for file

Additional file 3 Additional figure 3. Chromatography of DSN on Sephacryl S-200. A Sephacryl S-200 column (1 × 70 cm) was equilibrated with 50 mM Tris-HCl, pH 7.15 containing 150 mM NaCl and eluted at a flow rate 0.3 ml/min. Fractions with DNAse activity are indicated in grey. Click here for file
