# Supplementary figures and images for: Isolation, characterization and molecular cloning of Duplex-Specific Nuclease from the hepatopancreas of the Kamchatka crab
Source: BMC Biochem. 2008 May 21;9:14. doi: 10.1186/1471-2091-9-14 (PMC2413221; doi:10.1186/1471-2091-9-14)

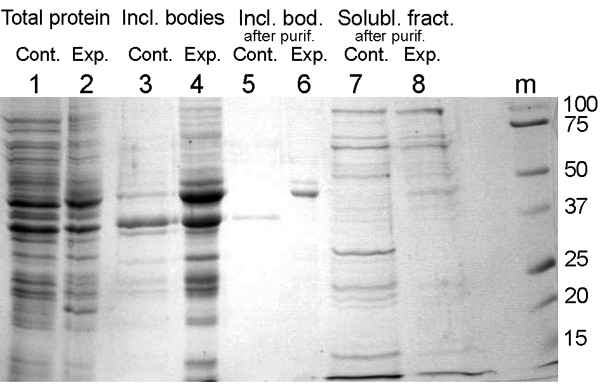

Supplement: Additional file 1 — Additional figure 1. DSN expression in E. coli. SDS-PAGE of protein fractions before and after purification by metal-affinity chromatography. Cont lanes: protein samples obtained from control E. coli strains containing pET; Exp lanes: proteins obtained from E. coli strains expressing pET-DSN. Lane 1 and 2, total protein; lane 3 and 4, protein compositions of inclusion bodies; lane 5 and 6, proteins purified from inclusion bodies by metal-affinity chromatography; lanes 7 and 8, proteins purified by metal-affinity chromatography of soluble fractions. M denotes the markers. Molecular masses of standards are indicated at left. [file 1471-2091-9-14-S1.tiff]
